# Densification of Magnesium Aluminate Spinel Using Manganese and Cobalt Fluoride as Sintering Aids

**DOI:** 10.3390/ma13010102

**Published:** 2019-12-24

**Authors:** Ali Talimian, Vaclav Pouchly, Karel Maca, Dusan Galusek

**Affiliations:** 1 Centre for Functional and Surface Functionalised Glass, Alexander Dubcek University of Trencin, 91150 Trencin, Slovakia; ali.talimian@tnuni.sk; 2 CEITEC BUT, Brno University of Technology, Purkynova 123, 62100 Brno, Czech Republic; vaclav.pouchly@ceitec.vutbr.cz (V.P.); maca@fme.vutbr.cz (K.M.); 3 Faculty of Mechanical Engineering, Brno University of Technology, Technicka 2, 62100 Brno, Czech Republic; 4 Joint Glass Centre of the IIC SAS, TnUAD and FChPT STU, 91150 Trencin, Slovakia

**Keywords:** MgAl_2_O_4_, lithium fluoride, cobalt fluoride, manganese fluoride, spark plasma sintering, grain growth

## Abstract

Highly dense magnesium aluminate spinel bodies are usually fabricated using pressure-assisted methods, such as spark plasma sintering (SPS), in the presence of lithium fluoride as a sintering aid. The present work investigates whether the addition of transition metal fluorides promotes the sintering of MgAl_2_O_4_ bodies during SPS. At the same time, such fluorides can act as a source of optically active dopants. A commercial MgAl_2_O_4_ was mixed with 0.5 wt% of LiF, MnF_2_, and CoF_2_ and, afterwards, consolidated using SPS at 1400 °C. Although MnF_2_ and CoF_2_ promote the densification as effectively as LiF, they cause significant grain growth.

## 1. Introduction

Magnesium aluminate spinel is a material of interest for optical applications due to its excellent mechanical and optical properties. MgAl_2_O_4_ has low density (3.58 g cm^−3^), typical fracture toughness of 1.9 MPa·m^0.5^, and high optical transmissivity in the visible to mid-infrared ranges [1,2,3,4,5,6,7]. Moreover, the spinel structure can host optically active elements, e.g., transition metal ions [8,9,10]. Having a symmetrical-cubic structure, transparent MgAl_2_O_4_ ceramics with high optical homogeneity can be fabricated by removing the scattering centers, such as pores and impurities [7,11,12,13]. Fabricating highly dense MgAl_2_O_4_ is, however, difficult because of the slow diffusion of oxygen. Therefore, spinel is usually densified by two-stage sintering, i.e., pressure-less sintering followed by hot isostatic pressing (post HIPing). Alternatively, spinel can be produced via single-stage pressure-assisted sintering, such as hot pressing (HP) or spark plasma sintering (SPS) [2,14,15,16,17]. Using the SPS method makes it possible to fabricate highly dense spinel bodies at a significantly lower temperature and a shorter time as compared with the other methods; this enables suppressing grain growth and producing high-quality samples.

Lithium fluoride (LiF) is a conventional sintering aid in processing MgAl_2_O_4_; it promotes the densification by producing transient liquid at low temperatures and introducing cation defects into the spinel structure. Moreover, LiF removes carbon contamination by forming volatile CF_x_ species. [18,19,20,21,22]. However, lithium incorporation into the MgAl_2_O_4_ structure can have a detrimental effect on optical properties, especially when spinel is doped with optically active elements [22,23].

Transition metal fluorides that melt at low temperatures are other suitable candidates to be used as additives for sintering of magnesium aluminate spinel. Such dopant provides double benefits. They assist densification through the formation of a transient liquid and, at the same time, introduce an optically active element into the MgAl_2_O_4_ structure. In this study, magnesium aluminate spinel bodies were fabricated by spark plasma sintering of a commercial aluminate spinel powder using LiF, MnF_2_, and CoF_2_ as sintering additives. The effect of sintering aids on densification behavior and final microstructure was investigated.

## 2. Materials and Methods

A commercial magnesium aluminate spinel powder, Baikalox S30CR (Baikowski, Paris, France) was used as the starting material in this study. The powder is characterized by a BET specific surface area of 26 m^2^g^−1^ and a median particle size (d_50_) of 0.2 µm according to the data provided by the supplier. The spinel powder contains minute amounts of impurities, mainly, S(600), Na(41), and Ca(15) in wt. ppm. Lithium fluoride (LiF), manganese fluoride (MnF_2_), and cobalt fluoride (CoF_2_), ACS grade > 99.0, purchased from Sigma-Aldrich (St. Louis, MO, USA) were used as sintering aids.

MgAl_2_O_4_ ceramics doped with the sintering aids (0.5 wt%) were prepared by dispersing and mixing of powders in isopropanol, ACS grade >99.0%, using an ultrasonic homogenizer (UW2200, BANDELIN, Berlin, Germany). Then, the mixtures were transferred to a rotary evaporator and dried. Ready-to-press (RTP) powder was prepared by passing the dried mixture through a sieve with a screen mesh of 500 µm.

Samples were consolidated using a spark plasma sintering machine (Dr. SINTER SPS-625, FUJI, Tokyo, Japan). The RTP powder was filled in a graphite die with an inner diameter of ca. 12 mm. The powder was separated from the die by graphite paper placed between powder, punches, and the die wall. The die was then wrapped in a carbon felt and placed between the moving rams of the SPS. The sintering schedules consisted of fast increases of the temperature to 600 °C in 3 min followed by heating of the sample at a constant heating rate of 100 °C min^−1^ to 1400 °C at which the shrinkage stops; therefore, sintering processes were carried out with no dwelling time to avoid unnecessary grain growth. The sintering was carried out under vacuum (5 to 9 Pa). A constant uniaxial pressure of 75 MPa was applied above 800 °C.

The displacement of punches and the temperature were recorded during the whole heating/cooling step. The pellets’ temperature was measured constantly by using an optical pyrometer focused on the hole drilled into the die wall. The coefficient of thermal expansion (CTE) of the system (e.g., graphite die, paper, and punches) was determined separately (i.e., in a run without the specimen) throughout the temperature range of this study (600 to 1400 °C) in order to account for the instrumental error. The sintered pellets were subsequently subjected to a heat treatment at 800 °C (heating rate 2.5 °C min^−1^) for 60 min in air in a muffle furnace to remove the residual carbon from the surfaces.

The bulk density and apparent porosity of sintered bodies were measured using Archimedes’ method in deionized water according to the ASTM standard (C329-88(2016)) [24]. All provided values are the means of at least 10 independent measurements.

The melting temperature of sintering aids and their reactions with the spinel powder were studied using thermal analysis. The measurements were performed by a simultaneous thermal analyzer (STA 449 F1 Jupiter, Netzsch, Selb, Germany) in Differential Thermal Analysis, DTA, configuration, using alumina crucibles in flowing N_2_ (20 mL min^−1^). Thermal Gravimetric analysis, TG, was performed simultaneously. Data were collected on ca. 100 mg of mixtures containing 10 wt% of sintering aids upon heating at a constant rate of 20 °C min^−1^ to 1350 °C.

The samples’ microstructure was examined using a scanning electron microscope, SEM, (JEOL 7600F, JEOL, Tokyo, Japan) equipped with an energy dispersive X-ray spectrometer (EDXS, Oxford Instruments, Abingdon, UK). Small fragments were collected from the fractured surface of samples and fixed on aluminium sample holders using conductive adhesive tape and coated with carbon to prevent charging.

## 3. Results

**Figure 1:**
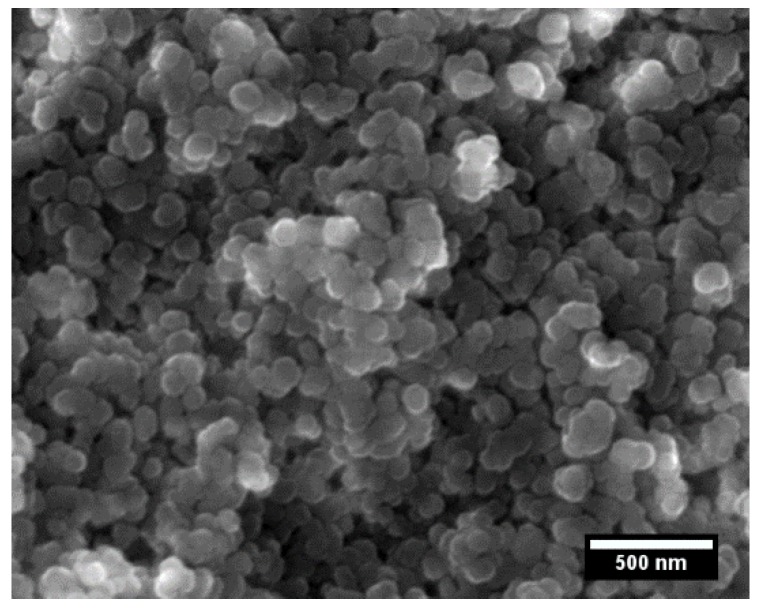
Scanning electron microscope (SEM) image of magnesium aluminate spinel powder.

Figure 1 shows the SEM micrograph of the magnesium aluminate spinel powder; the powder consists of submicron agglomerates comprised of smaller nanoparticles with a median diameter of 90 ± 15 nm. However, the specific surface area indicates a somewhat smaller primary particle size of approximately 64 nm. Similarly, Maca et al. examined the primary particle size of the same commercial MgAl_2_O_4_ powder, and reported an average particle size of 58 nm, by assuming that the primary particles have a spherical shape. The median particle size provided by the producer is, therefore, related to the size of agglomerates [25].

Table 1 summarizes the measured density and porosity of samples produced from the powder mixture containing 0.5 wt% of additives and the additive-free sample; the theoretical density of samples was calculated using the density of magnesium aluminate spinel (3.58 g cm^−1^) and the density of a respective additive following the rule of mixtures. The density of LiF, MnF_2_, and CoF_2_ are 2.64, 3.98, and 2.70 g cm^−3^, respectively. The measured density of an additive containing samples is within the range of experimental error comparable to the density of additive-free samples. While the residual porosity of additive-free samples is almost zero, the doped samples are characterized by limited amounts of closed porosity. Such behavior can be related to the evaporation of additives at high temperatures.

**Figure 2:**
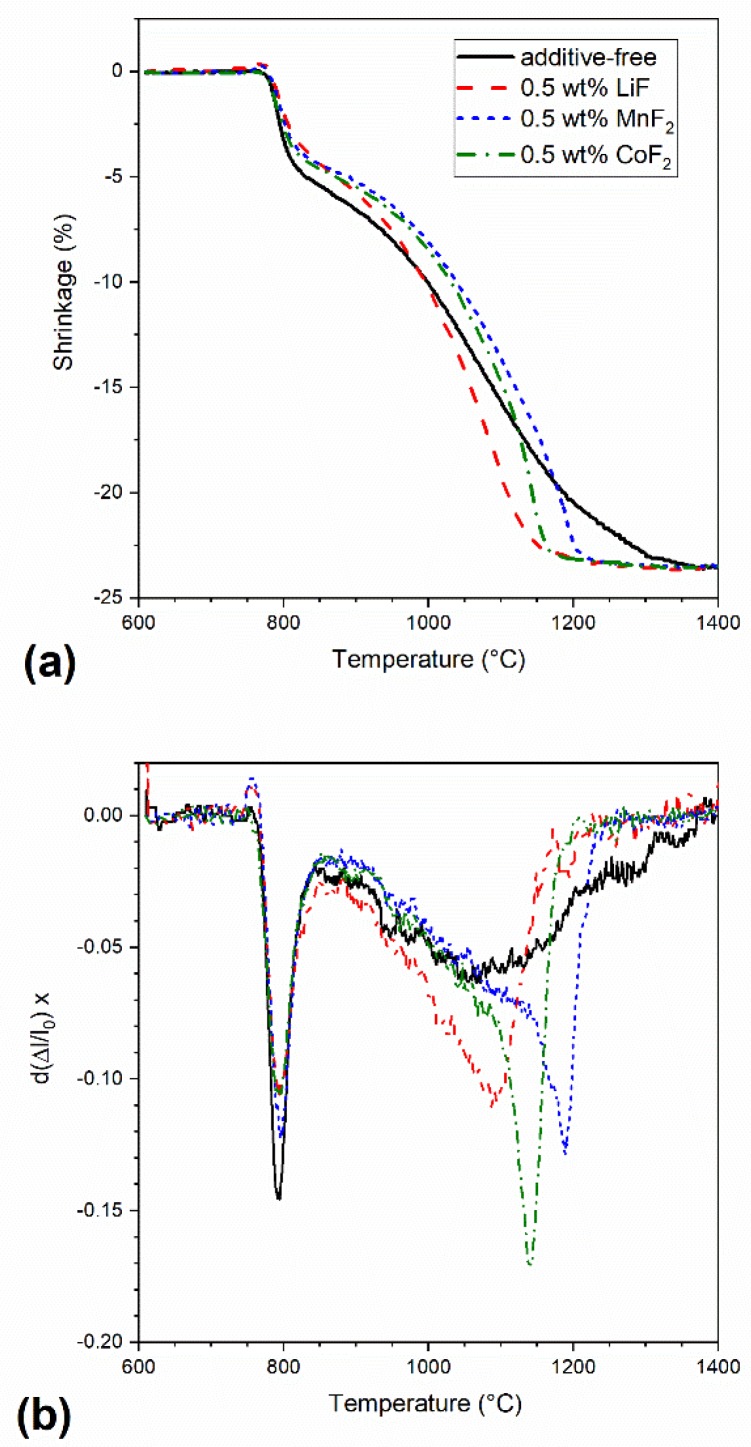
(**a**) Relative shrinkage of additive-free spinel and spinels doped with LiF, MnF_2_, and CoF_2_ (0.5 wt%) with temperature and (**b**) first derivative of the shrinkage calculated using Equation (1).

Figure 2 shows the temperature dependence of shrinkage and shrinkage rates of the powder mixtures containing 0.5 wt% of the additives and of the additive-free spinel powder during SPS; the shrinkage was determined by measuring the punch displacement upon heating at the constant heating rate of 100 °C min^−1^, between 600 °C and 1400 °C. The shrinkage rate was calculated point-by-point, using Equation (1):

(1) $$\frac{d\left(\frac{\Delta l}{l_{0}}\right)}{dt}=\frac{1}{l_{0}}\frac{\Delta l_{T+\delta T}-\Delta l_{T-\delta T}}{2\delta T}\dot{T}$$

where Δ*l* represents linear shrinkage measured at the temperature *T*, *l*_o_ is the original length of the sample, *t* represents time, and the variable $\dot{T}$ stands for heating rate. The shrinkage curves are characterized by two main regions, a rapid shrinkage of ~5%, occurring around 800 °C, followed by continuous shrinkage up to ~23%, after which the curve reaches a plateau. While the latter is related to the densification by sintering, the former is attributed to the powder particles’ rearrangement when pressure was applied [26]. The densification of all samples starts at around 850 °C. The sintering aids clearly decrease the temperature at which the densification is completed. The shrinkage curve of additive-free spinel reaches a plateau indicating the end of shrinkage, at 1350 °C, whereas the shrinkage of samples doped with LiF, CoF, and MnF_2_ stops at 1170, 1195, and 1250 °C, respectively. Moreover, the shrinkage rate of doped samples is significantly higher than that of the pure spinel, particularly at temperatures higher than 1000 °C (Figure 2b).

**Figure 3:**
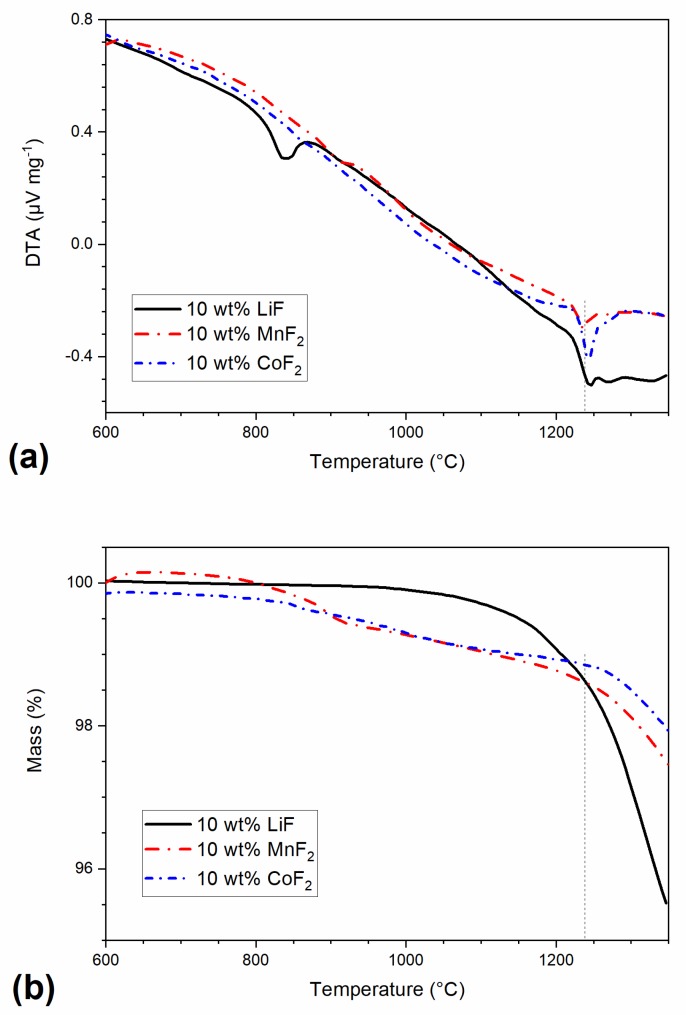
DTA (**a**) and TG (**b**) records of samples comprising 10 wt% of LiF, MnF_2_, and CoF_2_, heating rate = 20 °C min^−1^. Vertical line at 1230 °C represents onset of melting for TM fluorides doped spinel ceramics.

Figure 3 summarizes the results of DTA and TG analyses of powder mixtures containing 10 wt% of fluorides in the temperature interval between 600 to 1350 °C. These were carried out as reference measurements elucidating thermal processes on fluoride doped powders. The DTA curve of LiF-doped samples is characterized by a sharp endothermic peak at ~830° attributed to chemical reactions and melting of lithium fluoride, as discussed below (Equations (6) and (7)). In contrast, the sample containing MnF_2_ exhibits no clear endothermic effect at the melting temperature of MnF_2_ (856 °C). The behavior of the CoF_2_ containing sample is similar, showing no thermal effect, which could be attributed to melting of CoF_2_ (i.e., at ca 930 °C). However, all samples exhibit an endothermic peak at 1240 °C, attributed to the eutectic melting of magnesium fluoride, indicating chemical reactions between sintering aids and MgAl_2_O_4_, yielding MgF_2_, as pointed out in the following text. The TG curves show that the weight of LiF samples decreases rapidly above 1050 °C, while samples containing MnF_2_ and CoF_2_ exhibit slower weight loss in the following two steps: a slow decline above 850 °C followed by a rapid decrease over 1050 °C. The onset of weight loss can be correlated with the small endothermic effect on DTS curves associated with melting of MgF_2_ (1263 °C). The observed weight loss was then associated with evaporation of MgF_2_ from the melt. On the basis of the literature data, vapor pressure of molten MgF_2_ reaches ~13 Pa at 1270 °C and ~130 Pa at 1434 °C, so its loss is expected to be significant.

**Figure 4:**
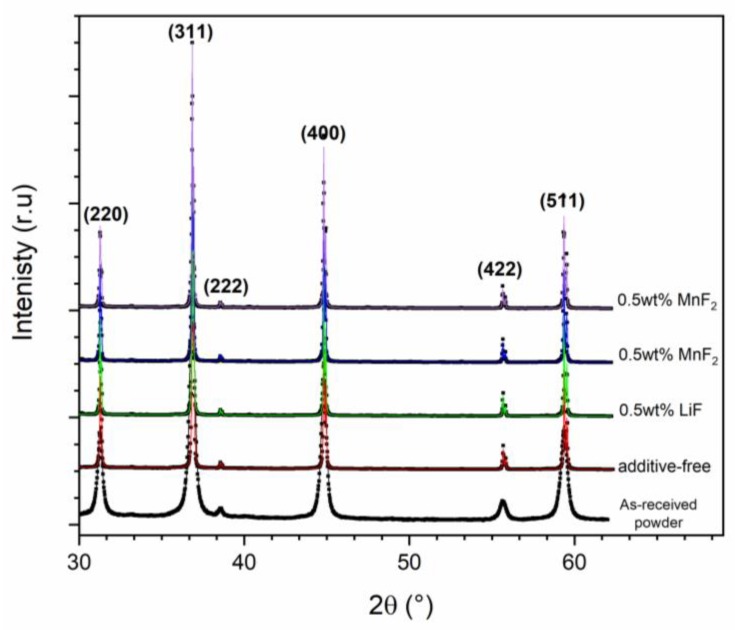
XRD patterns of additive-free and doped samples with 0.5 wt% (LiF, MnF_2_, and CoF_2_), spark plasma sintered at 1400 °C. The diffraction pattern of additive-free powder is also shown for comparison. The experimental data are fitted by the model patterns obtained by Rietveld refinement of experimental data.

Figure 4 shows the X-ray diffraction pattern of pellets produced by SPS at 1400 °C; the XRD pattern of as-received spinel powder is also shown for comparison. According to the XRD experiments, magnesium aluminate spinel is the only crystalline phase present in the samples; the sintered samples are characterized by sharp and narrow diffraction maxima that imply the sintering procedure (heating up to 1400 °C with the heating rate of 100 °C min^−1^, with no dwell time) increases the size of coherently diffracting domains (crystallites). The XRD patterns were analyzed further by using Rietvel refinement [27,28]. The lattice parameter of additive-free spinel is estimated to be 8.0798 ± 0.0002 Å while the lattice parameter for the samples doped with LiF, MnF_2_, and CoF_2_ are 8.0814 ± 0.0001 Å, 8.0833 ± 0.0003 Å, and 8.0833 ± 0.0001 Å, respectively. The incorporation of dopants into the spinel structure results in a slight increase of the lattice parameter due to size mismatch of doping cations and Mg^2+^ and Al^3+^ in the spinel crystal lattice.

**Figure 5:**
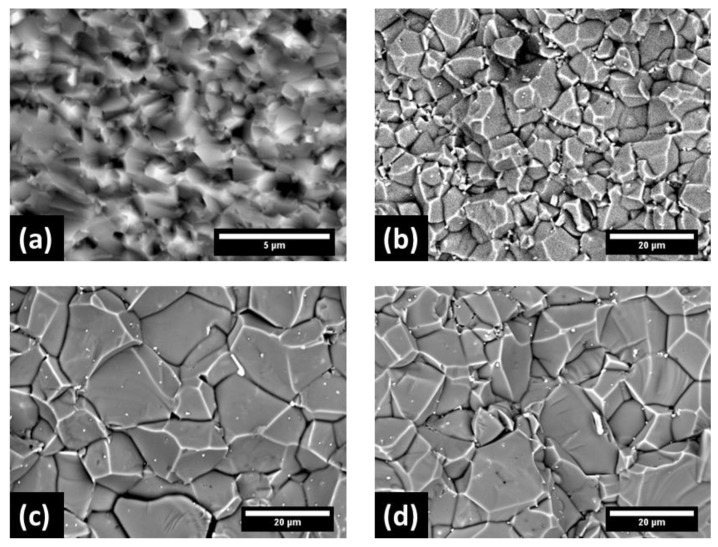
Scanning electron micrographs of the fracture surfaces after spark plasma sintering at 1400 °C. The images were recorded in a BSE mode from (**a**) additive-free sample, and the samples doped with 0.5 wt% of (**b**) LiF, (**c**) MnF, and (**d**) CoF_2_.

Figure 5 shows the fracture surface of additive-free and doped samples with the 0.5 wt% of LiF, MnF_2_, and CoF_2_. All doped samples exhibit significant grain coarsening. The LiF-doped spinel is, interestingly, characterized by a smaller grain size as compared with the spinel doped with MnF_2_ and CoF_2_.

**Figure 6:**
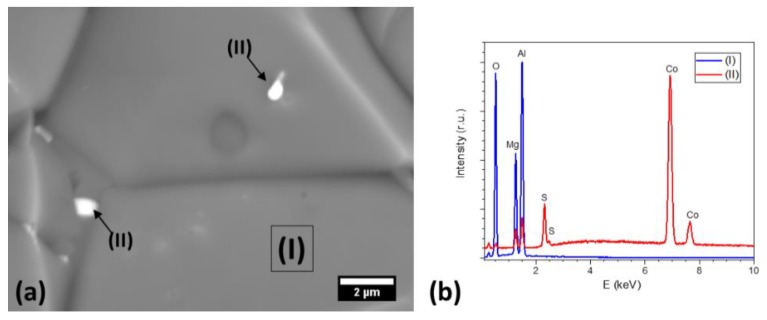
(**a**) Scanning electron micrograph showing in detail bright spots in CoF_2_ doped spinel after spark plasma sintering and (**b**) the EDX spectrum of the selected areas/spots: (I), spinel grain and (II), bright spot.

Bright spots observed on fracture surfaces were studied by EDX. Figure 6 shows a typical EDX spectrum collected from a bright spot in a CoF_2_ doped sample. The spots also contain, along with doping ions, a significant concentration of Sulphur, implying that the sulphate impurities in the spinel powder reacted with the dopant yielding sulfate phases during sintering. However, the content of sulphates was below the detection limit of X-ray diffraction, and the size of sulphate inclusions too small to be identified by EBSD.

## 4. Discussion

Figure 2 shows that the onset of densification of all studied samples occurs at a lower temperature than in conventional sintering. The application of pressure during spark plasma influences densification in two ways. First, powder particles rearrange under pressure. Secondly, the densification mechanism is also affected, due to grain boundary sliding [24,27]. Consequently, the maximum in densification rate of SPS samples is achieved at a lower temperature as compared with that reported for conventional sintering of the same powder (1350 °C, S30CR, Baikowski) [29,30].

The LiF-doping results in a higher densification rate, and larger grains than in the additive-free spinel are formed. The interaction between LiF and MgAl_2_O_4_ above the melting LiF point, 840 °C, and the formation of liquid are described as follows [31,32]:

(2) $$LiF\left(l\right)+MgAl_{2}O_{4}\left(s\right)\;\to \;LiF:MgF_{2}\left(l\right)+LiAlO_{2}\left(s\right)$$

The transient liquid enhances the densification through two main mechanisms, liquid redistribution facilitates particle rearrangement and the fluorine rich liquid enhances the mass transport. Moreover, lithium aluminate spinel can produce solid solution with magnesium aluminate spinel and introduces structural defects according to:

(3) $$LiAlO_{2}\;\overset{MgAl_{2}O_{4}}{\to }\;Li_{Mg}^{/}+V_{Al}^{///}+Al_{Al}^{\times }+2O_{O}^{\times }+2V_{O}^{{}^{..}}$$

Introduction of structural defects, such as oxygen vacancies, facilitates the movement of ions, particularly oxygen ions and, in turn, promotes the densification, as well as the grain growth. With the further increase of temperature, the evaporation rate of the transient liquid accelerates (above ca. 1100 °C, Figure 3b) and the liquid phase is effectively removed from the system. Consequently, the densification rate decreases.

(4) $$LiF:MgF_{2}\left(l\right)\;\to \;LiF\left(g\right)+MgF_{2}\left(g\right)$$

Finally, gaseous MgF_2_ reacts with LiAlO_2_ forming spinel again:

(5) $$2LiAlO_{2}\left(s\right)+MgF_{2}\left(g\right)\;\to \;2LiF\left(g\right)+MgAl_{2}O_{4}\left(s\right)$$

Considering densification behavior of MnF_2_ and CoF_2_ similar to that of the LiF doped samples, a similar sintering mechanism can also be expected for the transition metal fluorides. Surprisingly, although CoF_2_ and MnF_2_ have higher melting temperatures than LiF, the CoF_2_ and MnF_2_ doped spinel exhibits more extensive grain coarsening than the LiF doped ones.

Interestingly, the DTA records show no endothermic effect, which would indicate melting of pure transition metal fluoride additives around the expected temperatures of their melting. Only a small endothermic effect corresponding to melting of MgF_2_ implies chemical reactions between MnF_2_ or CoF_2_ and MgAl_2_O_4_ yielding transition phases. Due to the similar ionic radius of magnesium, manganese and cobalt ($r_{Mg^{2+}}=72\;\mathrm{pm},\;r_{Mn^{2+}}=70\;\mathrm{pm},\;\mathrm{and}\;r_{Co^{2+}}=75\;\mathrm{pm})$, it can be assumed that the transition metal ions replace magnesium ions in the spinel crystal lattice, producing MgF_2_:

(6) $$MnF_{2}\left(s\right)+MgAl_{2}O_{4}\left(s\right)\;\to \;MnAl_{2}O_{4}\left(s\right)+MgF_{2}\left(s\right)$$

(7) $$CoF_{2}\left(s\right)+MgAl_{2}O_{4}\left(s\right)\;\to \;CoAl_{2}O_{4}\left(s\right)+MgF_{2}(s)$$

The formation of a solid-solution with different divalent cations within the spinel structure results in spinel structure strain, as well as introduction of point defects, such as oxygen vacancies as a result of hosting divalent ions in octahedral sites. [7,33]. The TG results, Figure 3b, confirm that the weight loss of MnF_2_ and CoF_2_ doped samples begins at higher temperatures than in the LiF doped material (1250 °C vs. 1100 °C). The transient liquid is, therefore, present at grain boundaries for a longer time, providing a faster diffusion path for the elements, and resulting not only in efficient densification but also in more pronounced grain growth. This resulted in the increase of the median grain size from 0.8 µm in undoped ceramic to 10.3 µm, 14.0 µm, and 11.6 µm in LiF, MnF_2_, and CoF_2_ doped spinel, respectively, as shown in Figure 5. Apart from the presence of transient liquid in spinels doped by transition metal fluorides, the finer grain size of LiF doped samples can be attributed also to Zener pinning effect of LiAlO_2_ (Equation (1)) precipitated at grain boundaries [34].

Transition metal fluorides act as sintering aid during the densification of magnesium aluminate spinel and produce spinel structures containing optically active ions, e.g., Mn^2+^ and Co^2+^, that can be used in applications such as white LEDs or Q-switches. [35,36] Further studies are required to evaluate whether and how the addition of transition metal fluorides affects the densification and final properties of magnesium aluminate spinel ceramics.

## 5. Conclusions

Highly dense magnesium aluminate spinel bodies doped with LiF, MnF_2_, and CoF_2_ were produced using spark plasma sintering. Although the contribution of CoF_2_ and MnF_2_ to the densification of MgAl_2_O_4_ is more complicated as compared with LiF, they promote the densification almost as efficiently as LiF, despite the higher melting points of transition metal fluorides. The MnF_2_ and CoF_2_ containing samples exhibit larger grains as compared with LiF-doped spinel spark plasma sintered under the same conditions.

## Figures and Tables

**Table 1:** Relative density and apparent porosity of additive-free and transition metal fluoride-doped samples produced by spark plasma sintering (SPS) at 1400 °C (no isothermal dwell). The numbers in parenthesis represent standard errors.

Sample	Relative Density (%)	Apparent Porosity (%)
Additive-free	99.90 (0.02)	0.07 (0.03)
0.5 wt% LiF	99.6 (0.1)	0.43 (0.00)
0.5 wt% MnF_2_	99.4 (0.7)	0.57 (0.06)
0.5 wt% CoF_2_	99.7 (0.2)	0.33(0.07)
